# Implementation Mapping to Identify Best Practices for Implementing Population-Wide Genomic Screening Programs: Protocol for the FOCUS (Facilitating the Implementation of Population-Wide Genomic Screening) Study

**DOI:** 10.2196/73718

**Published:** 2025-10-07

**Authors:** Megan Roberts, Jarrod Marable, Kimberly Foss, Cason Whitcomb, Deborah Cragun, Adam Buchanan, Miranda Hallquist, Nathaniel Baker, Rebecca Bosch, Derek W Craig, Ingrid Wagner, Maria Fernandez, Chanita Hughes-Halbert, Caitlin Allen

**Affiliations:** 1 University of North Carolina Hospitals, Chapel Hill, NC, United States; 2 Department of Implementation, Wake Forest University School of Medicine, 525 Vine Street, Winston-Salem, NC, 27101, United States; 3 University of South Florida, Tampa, FL, United States; 4 Geisinger Health System, Danville, PA, United States; 5 Department of Public Health Sciences, College of Medicine, Medical University of South Carolina, Charleston, SC, United States; 6 The University of Texas Health Science Center at Houston, Houston, TX, United States; 7 University of Southern California, Los Angeles, CA, United States

**Keywords:** protocol, learning health system, population genomic screening, health equity, implementation science

## Abstract

**Background:**

Population-wide genomic screening (PGS) for genetic conditions such as hereditary breast and ovarian cancer syndrome, Lynch syndrome, and familial hypercholesterolemia presents opportunities to reduce morbidity and mortality among the 1%‐2% of the population at elevated risk for these serious, preventable diseases. With decreasing sequencing costs and growing support from national bodies, there are increasing numbers of PGS programs in the United States. However, guidelines and strategies to support implementation are limited, especially regarding equitable access to PGS. Contextual factors, such as organizational structures and processes, impact PGS implementation, often failing to benefit underrepresented populations. To address these challenges, we are completing the Facilitating the Implementation of Population-wide Genomic Screening (FOCUS) project, which will develop and test a freely available, web-based implementation toolkit to guide best practices for implementing PGS.

**Objective:**

The FOCUS project aims to (1) examine barriers and facilitators of PGS implementation at diverse health systems, (2) develop implementation strategies with input from an advisory panel and package them into the FOCUS toolkit, and (3) evaluate the toolkit’s impact on improving PGS reach, effectiveness, adoption, and maintenance using a hybrid stepped-wedge cluster randomized trial design.

**Methods:**

We will complete implementation mapping, guided by the Consolidated Framework for Implementation Research integrated with health equity, and the Reach, Effectiveness, Adoption, Implementation, and Maintenance framework for Health Equity to develop and evaluate an equity-focused PGS implementation toolkit. The study will involve 10 design sites to identify implementation barriers and facilitators and 12 Test Sites to assess the toolkit’s effectiveness. Both design and test sites will be representative of the following 4 stages of implementation: exploration or emerging, planning, implementation, and sustainment.

**Results:**

The FOCUS project was funded in September 2024 and will conclude in June 2029. The project was funded through the Advancing Genomic Medicine Research Program at the National Human Genome Research Institute (R01HG013851-01). Data collection for aim 1 (qualitative interviews with implementation team members, patients, and laboratory vendors) began January 2024. At the time of reporting, 33 interviews have been completed with implementation team members, 8 with patients, and two with laboratory vendors. Qualitative analyses for aim 1 are underway at the time of reporting.

**Conclusions:**

The FOCUS toolkit will establish a standardized approach to scaling PGS programs across diverse populations and settings, ensuring genomics benefits are accessible to all.

## Introduction

### Background

Population genomic screening (PGS) offers great promise in identifying the 1%‐2% of the population that carries a pathogenic variant that puts them at elevated risk for serious, yet manageable genetic conditions such as hereditary breast and ovarian cancer syndrome, Lynch syndrome, and familial hypercholesterolemia [1-5]. Rapidly decreasing sequencing costs, endorsement of PGS by national bodies, and attention to precision medicine applications have accelerated the spread of PGS programs [6,7].

Despite this growing momentum, no clear guidelines or strategies exist to support implementation of PGS. Contextual factors, including program-level procedures, organizational structure, patient population characteristics, stakeholder engagement, service delivery models, and access to genetic counseling influence implementation of these large-scale PGS initiatives [8-11]. Given the anticipated growth of PGS programs, practice-based research is needed to better understand these and other factors to inform implementation of PGS programs across diverse settings [11].

Previous genomic-implementation research studies have used systematic approaches grounded in implementation science to develop, evaluate, and user test implementation strategies. These implementation strategies were packaged into a toolkit for Universal Tumor Screening for Lynch Syndrome, and the toolkit components were found to be acceptable, appropriate, and meet the needs of stakeholders [12,13]. Yet, there remains a critical gap in translating such implementation strategies to broader PGS programs, especially those aiming to serve underrepresented populations and reduce disparities in access to genomic services [14-19].

To address this gap, our study aims to facilitate growth of PGS programs and enhance recruitment and retention of representative populations by developing and testing a freely available, online, multicomponent implementation guide (FOCUS [Facilitating the Implementation of Population-wide Genomic Screening] toolkit) to identify best practices for PGS implementation. Our project’s specific aims are to (1) describe differences in processes, barriers, and facilitators to equitable implementation across sites at various stages of PGS implementation; (2) develop and package implementation strategies into the FOCUS toolkit to support the equitable implementation of PGS programs; (3) evaluate the impact of the FOCUS toolkit on improving implementation of PGS in diverse populations and settings using a hybrid stepped wedge cluster randomized trial. This project will establish a gold standard approach for equitably integrating PGS and offer the field generalizable methods and knowledge about PGS implementation. In this paper, we describe the planned approach for achieving these aims and highlight how this approach is intended to advance the goal of implementing equitable PGS programs that improve population health.

### Contributions to the Literature

1. This study addresses a critical gap in implementation science by developing and testing an implementation toolkit to guide best practices for implementing PGS.
2. By examining barriers and facilitators across diverse settings, this work identifies key contextual factors, such as organizational structures and processes, that influence equitable PGS implementation.
3. Through its hybrid stepped-wedge cluster randomized trial, the study advances methods for evaluating the scalability and sustainability of PGS programs.
4. The findings contribute actionable insights to support the adoption and maintenance of PGS initiatives in healthcare settings across the United States.

## Methods

### Ethical Considerations

All components of this project involving human participants are approved through Wake Forest University School of Medicine institutional review board protocol (IRB00130700). We follow the Standards for Reporting Implementation Studies for all aims.

### Overall Study Design and Conceptual Model

**Figure 1.**
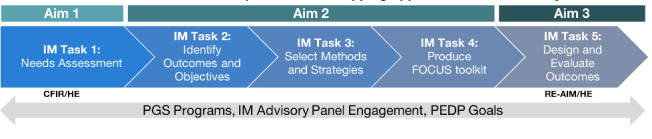
Overview of Facilitating the Implementation of Population-wide Genomic Screening (FOCUS) study: aims and corresponding implementation mapping tasks.

We will use an implementation mapping (IM) approach to complete the FOCUS project (Figure 1). Once an organization has made the decision to implement an evidence-based intervention (eg, PGS), use of IM supports the integration of this program in real-world settings [20]. IM is a structured framework involving five steps that incorporates theory, empirical evidence, and diverse perspectives with the target population to iteratively guide decision making at each step [20]. We incorporate a health equity (HE) focus in genomic medicine as described by the NHGRI in 2019 throughout all components of the IM process, the global applicability of genomic knowledge, fair and even access to genomic services such as testing and counseling, and unbiased implementation of genomic medicine. [17]

### Consolidated Framework for Implementation Research Integrated With the Health Equity Implementation Framework

The Consolidated Framework for Implementation Research integrated with health equity (CFIR/HE) helps create a robust approach for advancing equitable implementation. Consolidated Framework for Implementation Research integrated with health equity includes multilevel determinants of implementation and factors that inform differences in individuals’ access to health services, including key factors at the patient, provider, and system levels [21,22]. When combined with CFIR, the HE framework, which focuses on identifying and addressing structural and systemic barriers that perpetuate health disparities, supports a nuanced understanding of determinants of implementation. The CFIR/HE framework will be used to guide Task 1 of IM.

### Reach, Effectiveness, Adoption, Implementation, Maintenance Framework for Health Equity

The Reach, Effectiveness, Adoption, Implementation, and Maintenance framework for Health Equity (RE-AIM/HE) framework emphasizes the five dimensions of reach, effectiveness, adoption, implementation, and maintenance to evaluate implementation across diverse settings. Integration of HE adds an explicit focus on identifying and addressing health disparities by incorporating structural, cultural, and systematic factors that influence equitable implementation, which can help inform the evaluation and impact of new programs such as PGS across diverse populations and settings [23,24]. The RE-AIM/HE framework will be used to guide task 5 of IM.

### PGS Sites

We will work with 2 distinct sets of PGS sites: FOCUS design sites and FOCUS Test Sites. The FOCUS design sites will be part of Aim 1, which will involve identification and synthesis of evidence about the implementation processes, barriers, and facilitators for PGS implementation. We will include 10 diverse organizations that can speak to experiences across implementation stages: exploration, preparation, implementation, sustainment [25]. Exploration sites are defined as those sites that have not yet made a decision to adopt a PGS program and have not yet begun recruitment therein. Preparation sites are defined as those sites that have decided to implement a PGS program but have not yet begun actively recruiting participants. Implementation sites are defined as sites that have made the decision to adopt a PGS program and are actively recruiting participants but have not yet fully routinized and integrated PGS processes into clinical workflows. Finally, sustainment sites are defined as sites that are steadily recruiting participants and have routinized,integrated, or both types of PGS processes incorporated into their clinical workflows. We will also work with FOCUS Test Sites to rigorously evaluate the FOCUS toolkit for improving the implementation of PGS. These sites will also be at various stages of implementation. We plan to include 12 organizations as FOCUS test sites [25,26].

### IM Advisory Panel

Stakeholder engagement is a critical part of IM to ensure an equity-focused approach to the project and support all aspects of this work. Thus, we have organized an advisory panel of 13 individuals consisting of public health agency members, community members, patient groups, a private population screening company, primary care, and clinical stakeholders that will be engaged throughout each aim. The group will meet virtually on a quarterly basis and support the study team in prioritizing, designing, and conducting research throughout the duration of the project. Details about how the IM advisory panel will be engaged are included in each study aim.

### Study Aim 1: Describe Differences in Processes, Barriers, and Facilitators to Equitable Implementation Across Sites at Various Stages of PGS Implementation

The first Aim of the FOCUS study is aligned with IM Task 1. We will conduct a qualitative needs assessment among ten PGS programs (ie, FOCUS Design Sites) at various implementation stages (exploration, preparation, implementation, and sustainment). We will use the CFIR/HE framework to identify barriers and facilitators of implementing PGS [21,22]. These 10 sites will be enrolling either clinical or research PGS cohorts [24,27].

The needs assessment will be conducted via interviews of implementation team members (ITM), PGS patients, and laboratory sites. Participants will be assigned a study identification number and all identifying data elements such as names and dates will be removed. The questions asked during the interviews have been adapted from CFIR/HE and agreed upon by the FOCUS study team and the IM advisory panel. Interview questions will be specific to the type of participant, including implementation team members, patients, or laboratory site staff.

As interviews are completed, we will begin extracting qualitative data from the transcripts and audiovisual recordings, via Microsoft Teams for analysis. We will use rapid qualitative data analysis methods to code interviews for each site [28,29]. We will complete transcript summaries for each interview and match interview question responses to CFIR/HE domains and subdomains. These Transcript Summaries will be used in 2 subsequent qualitative data analysis steps—Process Mapping and Data Matrix Heat Mapping—that have been described by Salvati et al [30] Briefly, process maps will visually detail the PGS implementation process and workflow described by each team member during their interview. Upon completion of all interviews at the respective site, we will conduct member checking among these interviewees to reconcile discrepancies or omissions in the process maps. Member checking will include discussions with the original interviewees from each site to confirm our findings and process maps, allowing us to create a final aggregate process map for each site.

Site-level data matrix heat maps will be generated through an iterative process that involves mapping interview data to each of the CFIR/HE factors within a spreadsheet and then color coding each construct to represent the extent to which it serves as a facilitator or barrier for PGS implementation at that site. Site-level heat maps will then be consolidated and combined to allow for cross-site comparisons and the identification of patterns of factors that are similar and different across sites.

### Study Aim 2: Develop and Package Implementation Strategies Into the FOCUS Toolkit to Support the Equitable Implementation of PGS Programs

The second aim of the FOCUS study is to develop the FOCUS toolkit consisting of implementation strategies, materials, and protocols for implementing and improving PGS programs. This aim will cover IM Tasks 2‐4.

First, we will identify outcomes and objectives by working with the IM Advisory Panel to prioritize the facilitators, barriers, and inefficiencies identified in IM Task 1 by importance, relevance, and changeability. This will allow us to identify outcomes (ie, what needs to be added or changed to improve PGS implementation) and objectives (ie, how we will address the identified outcomes; IM Task 2).

We will work with the IM advisory panel during aim 2 to select evidence-based strategies to address the facilitators, barriers, and inefficiencies of PGS implementation (eg, information chunking and guided practice). Using the CFIR-Expert Recommendations for Implementing Change (ERIC) matching tool (IM Task 3) [29,31], which aligns ERIC strategies with CFIR factors identified in task 1. This will allow us to identify appropriate implementation strategies for each priority implementation need [32], including strategies that promote equity in access and quality of PGS. The study team will host a feedback session with the IM Advisory Panel on these potential methods and strategies at the conclusion of task 3 and integrate feedback from the panel based on this session.

Ultimately, we will draft implementation strategy protocols and develop materials to package our implementation strategies into the online FOCUS toolkit for PGS (IM task 4). Once the FOCUS toolkit draft has been developed, we will host a feedback session with the IM Advisory panel to capture and integrate recommendations. Finally, we will use a human-centered design approach to prototype and refine the FOCUS toolkit with our FOCUS design sites [33]. Engaging program champions and implementation team members from the 8 FOCUS design sites, we will conduct a live rapid prototyping and iterating session (nonstatistical approaches) to refine our proposed protocols and materials. Validated measures will be used to assess acceptability, appropriateness, and feasibility of using the FOCUS toolkit [34,35]. Through these live sessions, we will collect feedback until we have reached consensus among the IM advisory panel of the planned FOCUS toolkit materials. We will prioritize strategies that are common across PGS sites and that aim to achieve equity in PGS participation and implementation.

### Study Aim 3: Evaluate the Impact of the FOCUS Toolkit on Improving Implementation of PGS in Diverse Populations and Settings Using a Hybrid Stepped Wedge Cluster Randomized Trial

**Figure 2.**
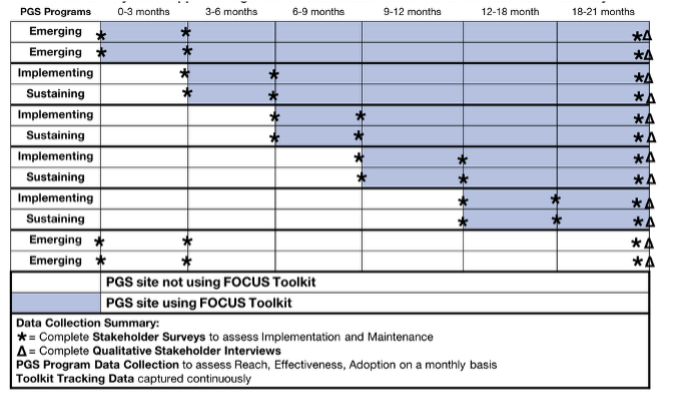
Hybrid stepped wedge cluster randomized trial with data collection summary.

The final aim of the FOCUS study is to evaluate the impact of the FOCUS toolkit on RE-AIM, for PGS in 12 FOCUS test sites with diverse populations and settings. The stepped-wedge cluster randomized trial design will evaluate the utility of the FOCUS toolkit for improving outcomes among the 12 FOCUS test sites [23]. This approach will allow us to assess improvements in RE-AIM/HE outcomes among sites at varying stages of implementation: exploration, preparation, implementation, and sustainment. Because sites in the exploration phase will not have begun implementing PGS before using the FOCUS toolkit (no baseline or pre-FOCUS toolkit data about the PGS program), they will be randomized 1:1 to either control (no FOCUS toolkit) or intervention (FOCUS toolkit) conditions using a parallel design. The 8 existing sites will be actively implementing or sustaining their PGS programs (have baseline or pre-FOCUS toolkit data) and will be randomized 1:1 to 1 of 4 possible time steps every 3 months for a 21-month implementation period (steps at 3, 6, 9, and 12 mo; Figure 2). This study is powered based on the effectiveness outcome measured by the proportion of individuals who have completed sample collection after being recruited for PGS. We will have 80% power with a type 1 error rate of 5% and an intracluster correlation coefficient of *P*=.01, to detect an increase in the proportion of participants submitting samples of at least 5.1% (10% before beginning to use FOCUS toolkit) to 6.0% (15% after beginning to use FOCUS toolkit). This hybrid stepped wedge cluster randomized trial design is a pragmatic approach that will allow us to simultaneously assess RE-AIM/HE outcomes across a representative and generalizable sample of PGS sites with varying amounts of baseline program implementation data and assessment of correlates of posttoolkit use outcomes [36-38].

Quantitative data about RE-AIM/HE outcomes will be collected using three tools: (1) PGS program data collection form, (2) stakeholder surveys, and (3) toolkit tracking data (Table 1). The PGS program data collection form will be completed monthly by each site using a REDCap (Research Electronic Data Capture; Vanderbilt University) form designed by the study team. The form will assess metrics related to the implementation outcomes of Reach, Effectiveness, and Adoption. Reach*,* or how well the PGS site recruits the intended audience, will be measured by the total number of people recruited to participate in the PGS program [39]. Effectiveness will be assessed based on how well the PGS program is achieving its intended public health outcome of identifying individuals with pathogenic variants in genes associated with tier 1 conditions. Our primary measure of effectiveness is the proportion of eligible individuals tested. We will also evaluate the rate of positive findings (number of positive results returned/number tested). Adoption will be assessed based on the number of providers who participate in the PGS program at each site out of the total number of providers eligible to participate at the site.

**Table 1.** Aim 3 outcome metrics.

Outcome metric	Measure and description	Data source	Level
Reach	Number of participants	EHR^a^ or site administrative records	Individual
Effectiveness	Positive findings: proportion of pathogenic variant and likely pathogenic variant results returned	EHR or site administrative records	Individual
Adoption	Number of providers participating in PGS^b^ program versus number of providers that are available at the institution	Administrative records	Site
Implementation	Acceptability, appropriateness, and feasibility	REDCap^c^ survey	Site
Maintenance	Intention to sustain PGS	REDCap survey [40]	Site

a electronic health record.

b PGS: population-wide genomic screening.

c REDCap: Research Electronic Data Capture.

The stakeholder surveys will be distributed to implementation stakeholders at each site before beginning to use the PGS toolkit, 3 months after starting to use the toolkit, and at the conclusion of using the toolkit. These will capture data to assess RE-AIM/HE outcomes of implementation and maintenance. Implementation assessment will include acceptability, appropriateness, and feasibility of PGS. These will be captured using the acceptability of intervention measure, intervention appropriateness measure, and feasibility of intervention measure, where the intervention is defined as PGS [35]. To assess maintenance, we will ask stakeholders to complete the program sustainability assessment tooprogram sustainability assessment tool to rate the PGS program on the presence of elements associated with long-term sustainability. We will also capture fidelity to the FOCUS toolkit through the toolkit tracking data, which will include website metrics of (1) FOCUS test sites’ registered users and (2) the number of times the FOCUS toolkit is opened, along with the number of pages or components visited.

Our primary research question is: does the use of the FOCUS toolkit improve PGS implementation and effectiveness? RE-AIM/HE outcomes among the 8 existing sites (ie, implementation and sustainment sites) that are actively implementing or sustaining PGS using generalized linear mixed models [41]. The primary temporal variable for the analysis of RE-AIM/HE outcomes is the time step; all participants enrolled during a given time step will be retained in that step regardless of study completion date. Statistical analysis will be based on the principle of intention to treat.

The step at which a participant was enrolled in the PGS program will be used as the treatment indicator. At the participant level, a mixed effects logistic regression model will be used to estimate the impact of sites’ use of the FOCUS toolkit strategies on the likelihood of participants returning for sample collection. Fixed effects of study time will be included in the model to adjust for secular temporal events throughout the study, and random site effects will be included to account for correlation of participants within sites over time. Participant-level baseline data will be assessed for association with study outcomes and when significant, included in adjusted models. Between-site sample size imbalance may impact the study power and estimation bias, thus initial models will be fit using Fay and Graubard bias-corrected sandwich variance estimator [42,43]. Period-specific recruitment totals will be assessed at the site level using a Gaussian distribution with an identity link. We will use generalized linear mixed models to assess the impact of the FOCUS toolkit among the 4 emerging sites that are part of the parallel design (FOCUS toolkit use vs no FOCUS toolkit use). In addition to addressing primary study outcomes, models including postintervention data from all sites will be assessed to determine if newly emerging PGS sites differ across outcomes as compared to existing sites. Generalized linear mixed effects regression will be assessed with an indicator of site implementation stage to assess postintervention RE-AIM/HE outcomes.

All RE-AIM/HE outcomes will also be assessed qualitatively through in-depth stakeholder interviews. At the conclusion of the implementation period, interviews will be conducted with 5 implementation stakeholders at each site (n=60): a program champion (n=1), a genetic counselor (n=1), an organizational leadership (n=1), and 2 members of the implementation team (n=2). Reach will include questions to understand whether all populations are equitably reached, who is not reached, and how the FOCUS toolkit supports better reach among diverse populations. Effectiveness will focus on whether program impact is equitable across groups, whether sample collection and findings are different across sociodemographics. Adoption will be assessed by asking about the uptake of PGS among clinicians and other key stakeholders within their setting. Implementation will be assessed by asking how PGS has been implemented, facilitators and barriers to implementation, and ways that the FOCUS toolkit helped the site overcome these barriers. To assess maintenance, we will ask stakeholders about the ways they plan to sustain the PGS program long-term and plans for institutionalization of the FOCUS toolkit [44].

A summary will be created immediately following each interview. These summaries will allow for high-level analysis during data collection, facilitate initial codebook development, and reduce the number of new codes requiring recoding during data analysis. Coding will be conducted using an a priori codebook guided by the RE-AIM/HE and CFIR (for contextual factors that are described as impacting RE-AIM/HE outcomes). After an initial review of this codebook, new codes will be added to the other relevant sections of the transcript text not fitting *a* priori codes. A team-based approach will be used to refine the codebook with discrepancies resolved by the study team. All coding will occur in MaxQDA and be completed by 2 coders. Qualitative and quantitative results will be reviewed together, and triangulation of data will occur using a rapid analytic coding matrix [45-47]. This will allow us to better understand how the PGS programs have changed over time and to what extent the FOCUS toolkit was adapted and used in accordance with the protocol.

## Results

The FOCUS project was funded in September 2024 and will conclude in June 2029. The project was funded through the Advancing Genomic Medicine Research Program at the National Human Genome Research Institute (R01HG013851-01). Data collection for aim 1 (qualitative interviews with implementation team members, patients, and laboratory vendors) began January 2024. At the time of reporting, 33 interviews have been completed with implementation team members, 8 with patients, and 2 with laboratory vendors. Qualitative analyses for aim 1 are underway at the time of reporting.

## Discussion

The FOCUS project will be the first to systematically identify facilitators and barriers to PGS implementation across a range of settings and stages of implementation. Further, the FOCUS toolkit will offer implementation strategies to address the identified barriers and leverage facilitators to support equitable PGS implementation. The diversity of PGS programs and comprehensive approach to data collection allows for findings to be broadly applicable and generalizable, recognizes contextual differences, collects data about strategies used in the real world, and is directly aligned with calls to develop a strategic PGS implementation agenda [48]. This approach can support widespread integration of PGS into routine practice by reducing evidence-based knowledge gaps about barriers and facilitators that impact PGS implementation.

There are several limitations to consider, which include potential dropout of FOCUS Design Sites and difficulty in recruiting diverse stakeholders and participants from each site. We have identified a program champion at each FOCUS design site and offered incentives for qualitative interviews. We will continue outreach until we reach saturation of qualitative themes (eg, new themes or concepts do not emerge) among PGS programs or until all associated stakeholders from each program have been approached. In addition, testing the toolkit with FOCUS test sites could be challenging. We will include sites at various stages of implementation (emerging, implementing, and sustaining). Given the timing, we will need to identify emerging sites closer to the start of aim 3 to ensure they are still in the emerging phase. As such, we will not identify these sites a priori; however, the literature has demonstrated a steep annual increase in the number of PGS programs in the United States, with triple the number of programs since 2017 [2,6,49]. Thus, it will be feasible to identify at least four emerging sites to participate. We will use our professional and research networks to identify and invite these sites to participate.

The FOCUS project will be foundational for the field by capturing what has been collectively learned to date about implementation of PGS and providing a flexible multicomponent implementation guide (FOCUS toolkit) to support the success of these programs with a special focus on advancing health equity in PGS. We will fill an unmet need to understand the best practices associated with implementation of PGS, and the toolkit that we produce will help develop, implement, and optimize PGS programs in the face of an evolving landscape. Findings from this research are critical to enhance the rapid expansion of PGS programs in the United States by providing generalizable methods for effective implementation. Ultimately, this will improve health outcomes through more comprehensive and equitable identification of individuals with inherited cancer and cardiovascular conditions.

## Abbreviations

CFIR/HE: Consolidated Framework for Implementation Research integrated with Health Equity
ERIC: Expert Recommendations for Implementing Change
FOCUS: Facilitating the Implementation of Population-wide Genomic Screening
HE: health equity
IM: implementation mapping
ITM: implementation team members
PGS: population-wide genomic screening
RE-AIM/HE: Reach, Effectiveness, Adoption, Implementation, and Maintenance framework for Health Equity
REDCap: Research Electronic Data Capture
